# The PDZ Protein Na^+^/H^+^ Exchanger Regulatory Factor-1 (NHERF1) Regulates Planar Cell Polarity and Motile Cilia Organization

**DOI:** 10.1371/journal.pone.0153144

**Published:** 2016-04-07

**Authors:** Anny Caceres Treat, David S. Wheeler, Donna B. Stolz, Michael Tsang, Peter A. Friedman, Guillermo Romero

**Affiliations:** 1 Laboratory for GPCR Biology, Department of Pharmacology & Chemical Biology, University of Pittsburgh, Pittsburgh School of Medicine, Pittsburgh, Pennsylvania, United States of America; 2 Medical Scientist Training Program, University of Pittsburgh School of Medicine, Pittsburgh, Pennsylvania, United States of America; 3 Department of Cell Biology, University of Pittsburgh School of Medicine, Pittsburgh, Pennsylvania, United States of America; 4 Department of Developmental Biology, University of Pittsburgh School of Medicine, Pittsburgh, Pennsylvania, United States of America; 5 Department of Structural Biology, University of Pittsburgh School of Medicine, Pittsburgh, Pennsylvania, United States of America; Texas A&M International University, UNITED STATES

## Abstract

Directional flow of the cerebrospinal fluid requires coordinated movement of the motile cilia of the ependymal epithelium that lines the cerebral ventricles. Here we report that mice lacking the Na^+^/H^+^ Exchanger Regulatory Factor 1 (NHERF1/*Slc9a3r1*, also known as EBP50) develop profound communicating hydrocephalus associated with fewer and disorganized ependymal cilia. Knockdown of NHERF1/*slc9a3r1* in zebrafish embryos also causes severe hydrocephalus of the hindbrain and impaired ciliogenesis in the otic vesicle. Ultrastructural analysis did not reveal defects in the shape or organization of individual cilia. Similar phenotypes have been described in animals with deficiencies in Wnt signaling and the Planar Cell Polarity (PCP) pathway. We show that NHERF1 binds the PCP core genes Frizzled (Fzd) and Vangl. We further show that NHERF1 assembles a ternary complex with Fzd4 and Vangl2 and promotes translocation of Vangl2 to the plasma membrane, in particular to the apical surface of ependymal cells. Taken together, these results strongly support an important role for NHERF1 in the regulation of PCP signaling and the development of functional motile cilia.

## Introduction

Ciliopathies constitute a growing class of genetic diseases with clinical manifestations that include neurodevelopmental defects, central nervous system (CNS) anomalies, laterality defects, and congenital heart disease [1]. Ciliary dysfunction resulting from one or more mutations in genes that regulate the assembly or function of primary, sensory, or motile cilia is commonly shared as the origin of these syndromes. Hydrocephalus is associated frequently with genetic ciliary dysfunction as a consequence of abnormalities in the ependyma, a layer of ciliated polarized epithelial cells that differentiate from radial glia to form the lining of the cerebral ventricles [2]. Mutations in genes involved in the assembly and structure of ependymal cilia affect cerebrospinal fluid (CSF) dynamics resulting in hydrocephalus [3–6]. The genetic factors that govern ciliary development and function in the ependyma remain poorly understood. However, recent work links ependymal ciliogenesis to non-canonical Wnt signaling, specifically to the Planar Cell Polarity (PCP) pathway [7, 8].

NHERF1 (EBP50/Slc9a3r1) is a member of the PSD-95/Discs-large/Zo-1 (PDZ) family of proteins [9]. NHERF1 contains two N-terminal PDZ domains and one C-terminal Ezrin/Radixin/Moesin/Merlin-binding domain (EBD) that attaches to the cytoskeleton [9]. Multiple functions of NHERF1 have been reported, including the organization of apical microvilli in polarized epithelium [10], the establishment of apical-basolateral polarity [11–13], and the scaffolding of signaling complexes [14–16]. Hydrocephalus was noted in NHERF1 knockout mice [17], but the origin of this phenotype has not been investigated. We report here an extensive characterization of the cause of hydrocephalus in NHERF1 knockout animals. We show that the phenotype is cross-species, since NHERF1/Slc9a3r1 deficiency causes hydrocephalus both in mice and in zebrafish injected with *nherf1/slc9a3r1* antisense morpholinos. Furthermore, we demonstrate that the phenotype is associated with defective ciliogenesis in the NHERF1 knockout/knockdown animals. The structure of the cilia of NHERF1^-/-^ animals appears normal. However, they are disorganized, present in reduced numbers, and functionally defective. Our data further suggest that the origin of this phenotype is linked to altered Wnt/PCP signaling.

## Experimental Procedures

### Reagents and Materials

CHO-N10 cells, which express NHERF1 under tetracycline control, were developed in our lab from a parental CHO cell line from ATCC [18]. Primary antibodies for HA were purchased from Covance. Anti-Vangl2 antibodies were from Abcam. Anti-NHERF1 antibodies were purchased from Upstate Biotechnology. Anti-GFP antibodies were from Clontech. Secondary antibodies were purchased from Jackson Immunoreagents or from Thermo Fisher. X-tremeGENE HP transfection reagent was purchased from Roche. Opti-MEM and Ham’s F-12 media were purchased from Life Technologies. All other reagents used were purchased from Sigma. HA-tagged human Fzd4 was kindly provided by Dr. T. Kirchhausen. HA-tagged rat Fzd1 was a generous gift from Dr. R. Habas. Vangl2 was purchased from Addgene and subcloned downstream of EGFP. Vangl1 and Vangl1ΔPDZ were a gift from Dr. P. Gros. HA-Vangl2 was a gift from Dr. D. Ginty.

### Immunoprecipitation and immunoblot

CHO-N10 cells stably expressing Fzd4 were transiently transfected with EGFP-Vangl2 or empty vector. NHERF1 expression was induced with 50 ng/ml tetracycline and after 48 h the cells were lysed with RIPA buffer supplemented with protease inhibitors and incubated for ice for 15 min. Lysates were incubated overnight at 4°C with HA.11 monoclonal affinity matrix (Covance). Total lysates and immunoprecipitated protein were analyzed by SDS-polyacrylamide gels and transferred to Immobilon-P membranes. The blots were probed with the following specific antibodies: NHERF1 (Santa Cruz), GFP (Life Technologies), and HA.11 (Covance). All primary antibodies were used at a concentration of 1 μg/ml.

### Live cell imaging/Vangl2 localization

CHO-N10 cells were transfected with HA-Fzd1 and either EGFP-Vangl2 or EGFP-V521A-Vangl2. Twenty-four h post transfection cells were treated with vehicle or 50 ng/ml tetracycline to induce NHERF1 expression and incubated for another 24–48 hr. Live cells were decorated with Covance HA.11 primary antibody for 30 min at room temperature, rinsed with PBS and then incubated with 2 μg/ml goat anti-mouse TRITC conjugated secondary antibodies for 30 min. After rinsing, the cells were imaged using a confocal microscope. The cross-correlation of HA-Frizzled 1 and EGFP-Vangl2 or EGFP- V512A-Vangl2 was measured using ImageJ software.

### Live Cell Image Cross-Correlation Spectroscopy

CHO-N10 cells transfected with EGFP-Vangl2 and HA-Fzd4 were treated with 50 ng/ml tetracycline to induce NHERF1. Live cells were incubated with Alexafluor 594-conjugated Covance HA.11 antibodies (2 μg/ml) for 30 min at room temperature to label Fzd4 receptors at the plasma membrane. Cells were rinsed and then imaged using an Olympus Fluoview1000 confocal microscope. To measure the autocorrelation and cross-correlation functions, the microscope was carefully focused on the plasma membrane, and 100–200 frames of a small area (30x30 pixels) were obtained by continuous scanning at a rate of 30 ms/frame. The autocorrelation and cross-correlation functions of EGFP-Vangl2 and HA-Fzd4 were calculated using an ImageJ plugin specifically written for this purpose, as described elsewhere (see [15]).

### Fluorescence recovery after photobleaching (FRAP)

CHO-N10 cells were transfected with HA-Fzd4 and EGFP-Vangl2 and incubated with HA.11 anti-HA antibodies (Covance). A subset of cells from each group were incubated with 5 μg/mL goat anti-mouse secondary antibody (Thermo Fisher, NY) for 30 min at room temperature to immobilize Fzd4 receptors. All FRAP measurements were done focusing on the plasma membrane adjacent to the coverslip. Cells that expressed EGFP-Vangl2 at the plasma membrane were identified and circular regions of interest were selected and bleached with the 488-nm laser line using an Olympus Fluoview 1000 equipped with a SIM scanner. EGFP fluorescence recovery of the bleached area was recorded over time. The data were fitted to a single exponential decay using GraphPad Prism and the immobile fraction of each group was determined.

### Immunohistochemistry

All mice used in this study were derived from a knockout line initially developed by Shenolikar et al [17]. The animals were bred from heterozygote parents because of the low fertility of the homozygote NHERF1^-/-^ animals. The brains of 4–10 week old animals (NHERF^-/-^ and wild-type littermates) were fixed in 4% formalin for 48 h at 4°C, dehydrated, embedded in paraffin and sectioned into 5μm slices using a microtome. For immunohistochemistry, glass slide mounted slices were rehydrated by successive washes with xylene, 100% ethanol, 95% ethanol, 75% ethanol, and PBS. Antigen retrieval was performed by heating the slides in citrate buffer (56°C, 45 min). Samples were blocked with 5% BSA and exposed to primary antibody (1–2 μg/ml) at 4°C overnight. The slides were washed and incubated with the appropriate secondary antibody (dilution 1:1000) for 2 h at room temperature, further stained with DAPI for 5 min, washed briefly, and covered with a coverslip. All tissues were examined with an Olympus Fluoview 1000 confocal microscope.

### Transmission Electron Microscopy (TEM)

Brains were harvested and immersion-fixed in 2.5% glutaraldehyde overnight at 4°C. Brains were sectioned such that ependymal epithelium was revealed on a surface of the tissue slice. Following fixation, tissue was washed 3x in PBS then post-fixed in aqueous 1% OsO_4_, 1% K_3_Fe(CN)_6_ for 1 h. Following 3 PBS washes, the tissue was dehydrated through a graded series of 30–100% ethanol, 100% propylene oxide then infiltrated in 1:1 mixture of propylene oxide:Polybed 812 epoxy resin for 1 h. After several changes of 100% resin over 24 h, brain slices were embedded in molds, cured at 37°C overnight, followed by additional hardening at 65°C for two more days. Ultrathin (60 nm) sections of tissue were collected on copper grids, stained with 2% uranyl acetate in 50% methanol for 10 min, followed by 1% lead citrate for 7 min. Sections were imaged using a JEOL JEM 1210 transmission electron microscope at 80 kV fitted with a side-mount digital camera.

### Cilia orientation measurements

To quantitate the organization of the cilia, TEM images of the surface of the ependyma were examined and the angle between the surface of the cell. The longitudinal axis of individual basal bodies were recorded using ImageJ and averaged for all cilia in each field. The deviation from the average orientation was determined for each individual cilium as θ-θ_Average_. The variance (S) of each sample was used to quantitate the distribution of orientations. Quantitative comparisons between variances were done using Bartlett’s test.

### Scanning Electron Microscopy (SEM)

Mouse tissues were processed as for TEM above, but 1-mm thick longitudinal slices that reveal the surface epithelium were used. Tissue was processed up to the final 100% ethanol, then chemically dried using hexamethyldisilazane. Dried slices were mounted onto aluminum stubs, grounded with silver paint then sputter coated with 3.5 nm gold/palladium (Auto 108, Cressington, Watford, UK). Samples were viewed in a JEOL JSM-6330F scanning electron microscope (Peabody, MA) at 3 kV.

### Ciliary Function Assay

Fresh mouse brains were sectioned into 1 mm thick slices using a hand held slicer (Zivic Instruments) and mounted onto a glass-bottomed chamber filled with clear MEM. To measure ciliary function, 2 μl of a suspension of 1 μm fluorescein-tagged beads (Molecular Probes, Eugene, Oregon) were added to the observation space (the third ventricle) and the motion of the fluorescent beads was monitored using a fluorescence microscope equipped with a 20X water immersion objective. Time courses of up to 60 sec were recorded and bead velocity was determined using ImageJ. For tracheal tissue, the tracheae were excised, cut longitudinally and mounted with the tracheal epithelium exposed. The fluorescent beads were added over the exposed surface and bead motion was examined as described.

### *nherf1* Gene Knockdown in Zebrafish

Zebrafish were originally obtained from NIH, but have been bred at the University of Pittsburgh facilities for over 10 years. All zebrafish experiments were approved by the University of Pittsburgh Institutional Animal Care and Use Committee. Embryos were obtained from wild-type (AB*) through natural mating. Two specific *nherf1/slc9a3r1* antisense morpholinos were used: *(5’-CCTGAGGTCGCTGGACATTTT-3’)* (NHERF1-MO), which targets the AUG initiation codon, and *(5’-ATATATCTGAACTCACCTTGGAGCT-3’)* (Splice-MO), which targets the exon 1 splice junction. Scrambled morpholinos of identical global composition (Control-MO) were used as controls. All MO were designed and synthesized by GeneTools, LLC (Philomath, OR). MO doses ranging from 1–10ng were injected into the 1-cell stage embryos as previously described [19]. Embryos were incubated to the desired stage, then directly imaged under a Leica stereomicroscope and photographed using a digital camera. No differences were noted between the phenotypes of control-MO-injected and uninjected embryos.

### Euthanasia

Animals were euthanized as directed by the University of Pittsburgh’s guidelines. This implies: Tricaine methane sulfonate for Zebra fish and CO_2_ asphyxiation followed by decapitation for the mice,

### Statistical analysis

All experiments were repeated at least three times. GraphPad Prism or the SPSS statistical package were used for all statistical analyses.

## Results

### NHERF1 depletion causes hydrocephalus

**Fig 1:**
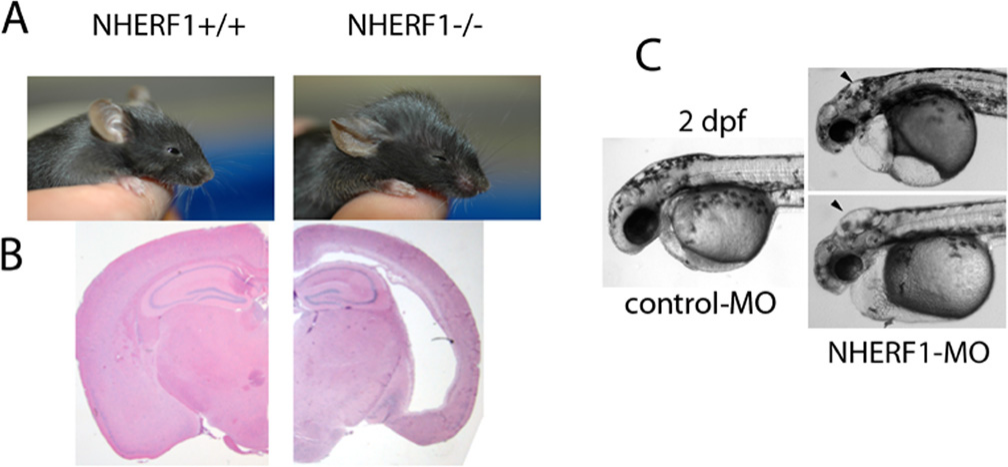
NHERF1^-/-^ depletion causes hydrocephalus. (A), Comparison of two 28-day old NHERF1^+/+^ (left) and NHERF1^-/-^ (right) littermates. (B) Nissl-stained coronal sections from NHERF1^+/+^ (left) and NHERF1^-/-^ mice at P28. (C) Depletion of *nherf1* in zebrafish embryos caused hydrocephalus (arrows) 2 days post-fertilization.

The mice used in these studies were derived from the NHERF1^-/-^ clones developed by Shenolikar et al [17] after back breeding with C57BL/6 for 10 generations to produce an isogenic line. About 35% of the NHERF1^-/-^ mice showed clinically relevant hydrocephalus (Fig 1*A* and 1*B*) between 28 and 32 days after birth. Ventricular dilation was detected in all animals examined (n = 12), independent of whether or not external signs of hydrocephaly were visible. Dilation of the cerebral ventricles was not found in any of the wild-type and in only one of the heterozygote animals. Detailed examination of brain slices from severely hydrocephalic NHERF1^-/-^ animals did not reveal obstructions in the ventricles, arachnoid granulations, or the cerebral aqueduct, suggesting that the syndrome is a form of communicating hydrocephalus. To confirm that NHERF1 knockdown is sufficient to cause hydrocephalus, we injected 1-cell stage zebrafish *(Danio rerio)* embryos with antisense morpholino oligonucleotides targeted to the *nherf1/slc9a3r1* initiation codon (NHERF1-MO). All injected embryos showed impaired balance and motility accompanied by severe hydrocephalus at 48 h post fertilization (hpf) (Fig 1*C*). To confirm the specificity of these effects, we injected embryos with a splice morpholino. Embryos injected with the splice-MO also developed hindbrain hydrocephalus, although the phenotype was not as severe (S1 Fig).

### NHERF1 depletion causes defective ciliogenesis

**Fig 2:**
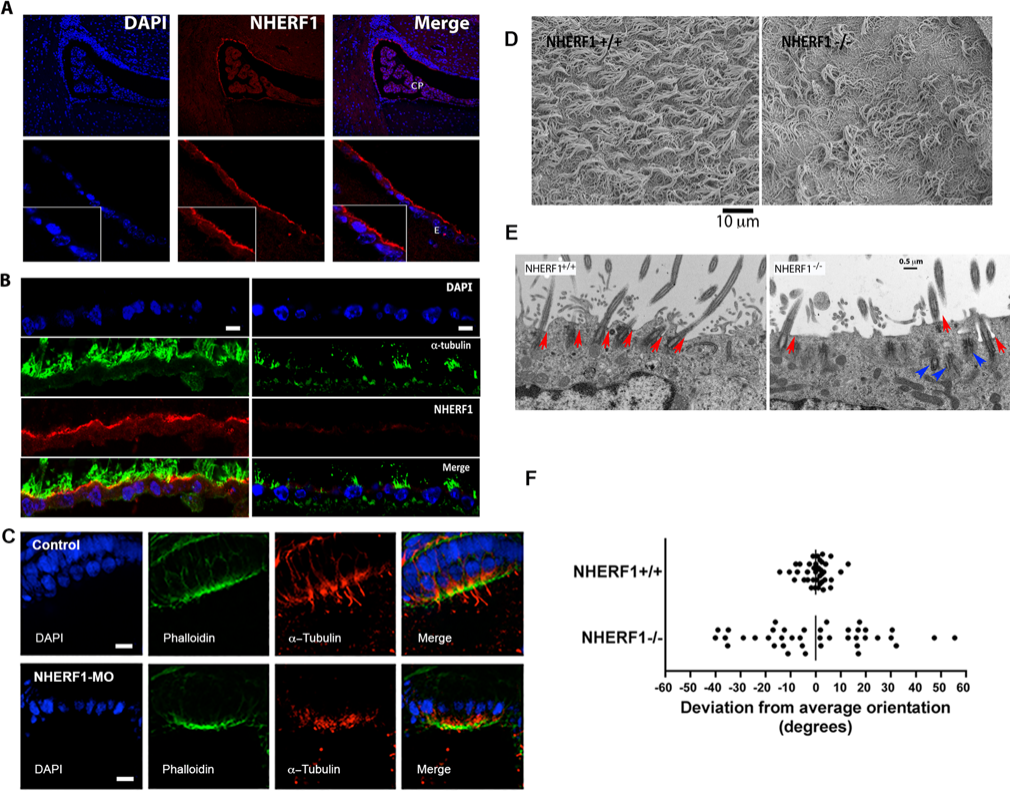
NHERF1 depletion causes defective ciliogenesis. (A) Immunofluorescence staining of a sagittal section of the third ventricle of a NHERF1^+/+^ mouse. CP: choroid plexus; E: ependymal layer. Sagittal sections of paraffin embedded P28 wild-type mouse brain were stained with anti-NHERF1 antibody followed by a TRITC-labeled secondary antibody. The sections were then imaged using a confocal microscope. (B) Ciliary defects in the NHERF1^-/-^ mouse. Sagittal sections of the brains of P28 wild-type and NHERF1^-/-^ mice were stained with antibodies specific for acetylated α-tubulin and NHERF1. The bar represents 10 μm. (C) Ciliary defects in the otic vesicle caused by injection of NHERF1-MO. Whole embryos (48 hpf) were fixed in 4% PFA, stained with phalloidin and anti-α-tubulin and the otic vesicles were examined by confocal microscopy. (D) Scanning electron micrograph of the ependyma of wild-type and NHERF1^-/-^ mice. (E) Defective orientation of ependymal cilia in the NHERF1^-/-^ mouse. Transmission electron micrographs of radial sections of the surface of ependymal cells from P28 wild-type and NHERF1^-/-^ mice. Red arrowheads: orientation of individual basal bodies. Blue arrowheads: embedded basal bodies. (F) Comparison of the orientation of ependymal cilia in NHERF1^+/+^ and NHERF1^-/-^ mice. Cilia orientation was determined from the angles formed by the basal body of each cilium and the tangent to the cell surface. The angles (θ) of all surface basal bodies within an image were measured using ImageJ and averaged for all cilia within each field. The deviation from the average orientation was determined for each individual cilium as θ-θ_Average_. The variance (S) of each sample was used to quantitate the distribution of orientations (S_wild-type_ = 29.03 (N = 38); S_knockout_ = 389.7 (N = 36)). Quantitative comparisons between both sets of samples were done using Bartlett’s test; the difference is significant with p<0.0001.

We examined the expression of NHERF1 in the ependyma and choroid plexus of wild-type mice. NHERF1 is abundantly expressed in the ependymal epithelium (Fig 2*A* and 2*B*). Because of the high levels of NHERF1 in ependyma, and given that impaired ciliary function is a frequent cause of hydrocephalus [3–6], we analyzed ependymal cilia by staining with acetylated α-tubulin antibodies (Fig 2*B*). Compared to wild-type animals, the number of cilia was reduced in NHERF1^-/-^ mice (Fig 2*B*). Many ependymal cells had fewer cilia, and some had none. A similar phenomenon was observed in the otic vesicles of zebrafish embryos injected with NHERF1-MO (Fig 2*C*). The otic vesicles of NHERF1-MO injected embryos, although normal in size (Fig 1*C*), contained very few cilia in 38 of the 42 specimens examined (Fig 2*C*). Importantly, NHERF1-MO injections did not disrupt the actin cap of the otic vesicle epithelium (Fig 2*C*), suggesting that NHERF1 knockdown had few effects on the apical-basolateral polarization of the otic vesicle cells. A similar phenotype was observed in the embryos injected with the splice-MO (S1 Fig), although fully developed cilia were observed in 28% of the splice-MO morphants. Scanning electron microscopy studies of mouse brains confirmed the tubulin staining data, demonstrating a significant reduction in the number of cilia in NHERF1^-/-^ mice. The images further showed significant differences in the orientation of the cilia, with the ciliary tufts of adjacent cells often pointing in opposite directions (Fig 2*D*). Importantly, the absence of NHERF1 did not alter the differentiation of ependymal cells as measured by the expression of characteristic markers such as S100β [2] (S2 Fig). To evaluate the orientation of the ependymal cilia, we performed transmission electron microscopy (TEM) experiments. Fig 2*E* compares two high-magnification images highlighting the relative orientation of the basal bodies (red arrows). Whereas the basal bodies of the wild-type animal form a single row just beneath the cell surface and point roughly in the same direction (Range: ± 11 degrees for the image shown in Fig 2*E*), the basal bodies of the knockout animals were scarce, less organized, located at different depths, and pointed in multiple directions (Range: ± 31 degrees for the shown image). The TEM images revealed numerous NHERF1^-/-^ cells devoid of cilia and others with basal bodies deeply embedded in the cytoplasm (Fig 2*E*; *blue arrowheads*). A very similar phenotype was described for the Celsr2^-/-^ mice [8, 20]. To obtain a quantitative representation of the data, we examined TEM micrographs from 6 P28 NHERF1^-/-^ animals and 3 wild-type littermates, and determined the fraction of basal bodies located at a distance greater than 500 nm from the plasma membrane, as described by Tissir et al [8]. A greater proportion of the basal bodies were entrapped in the cytosol of the NHERF1^-/-^ ependyma cells (38.6% vs 9.5%; p<0.0001). Most of the embedded basal bodies were not associated to cilia, although ectopic cilia were observed in some images. Fig 2*F* shows the distribution of the angles of the basal bodies from multiple TEM images obtained from 3 NHERF1^+/+^ and 3 NHERF1^-/-^ littermates. Whereas the basal bodies of the wild-type animals were roughly oriented in the same direction (± 11.9 degrees; N = 38), those of the NHERF1^-/-^ mice were highly disorganized, pointing in multiple directions (± 41.6 degrees; N = 36). To evaluate these differences quantitatively, we compared the variance of the distributions around the mean of the angles formed by the basal bodies and the tangent of the adjoining cell surface and analyzed the data using Bartlett’s test. The variances were significantly different (29.03, N = 38 vs. 389.7, N = 36 for the wild-type and knockout animals, respectively; p<0.0001). Embedded basal bodies were not included in these comparisons; therefore, these numbers underestimate the magnitude of the ciliary defects.

**Fig 3:**
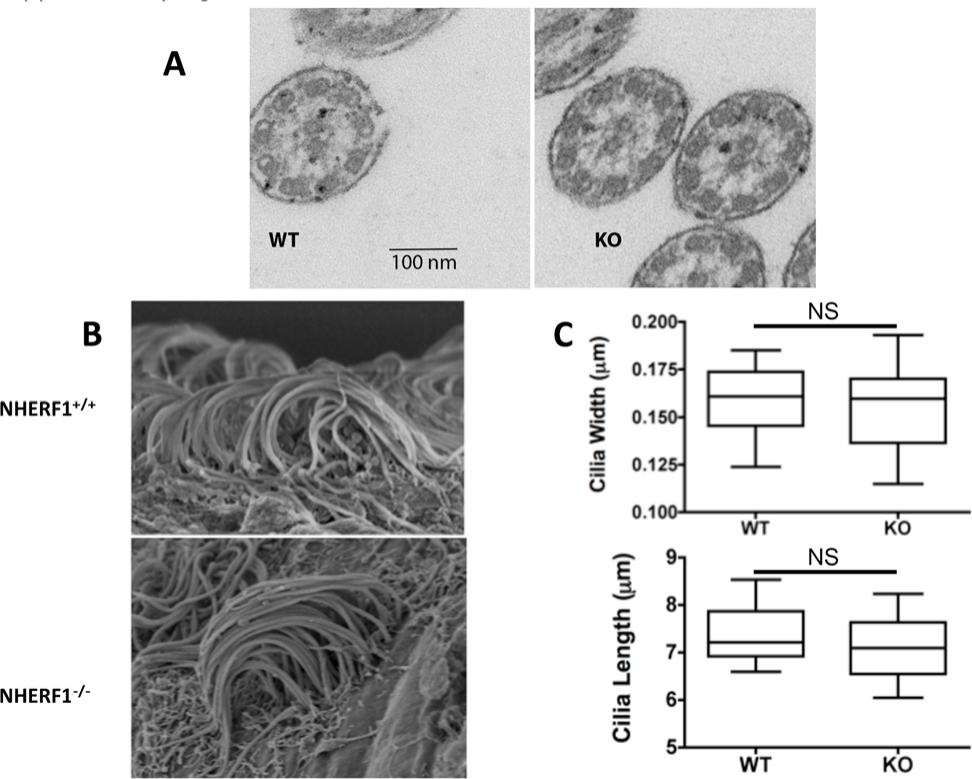
Ultrastructure of the ependymal cilia. (A) Transmission electron micrographs of the motile cilia of NHERF1^+/+^ and NHERF1^-/-^ does not reveal any differences in architecture. (B), (C), Scanning electron microscopy does not reveal any differences in length and diameter between NHERF1^+/+^ and NHERF1^-/-^ ependymal cilia. NS: the differences were not statistically significant.

The ultrastructure of the residual cilia found in NHERF1^-/-^ mice was unremarkable. The cilia displayed the typical 9+2 structure of normal motile cilia, and individual cilia were of the same length (7.12 ± 1.8 μm; N = 22) and diameter (0.16 ± 0.02 μm; N = 22) when compared to cilia of the wild-type littermates (length: 7.2 ± 1.4 μm; diameter: 0.162 ± 0.012 μm; N = 34) (Fig 3). This suggests that NHERF1 is not structurally involved in the architecture of motile cilia. We conclude that NHERF1 ablation causes reduced number and altered orientation of the cilia but does not change the structural organization of the cilium.

**Fig 4:**
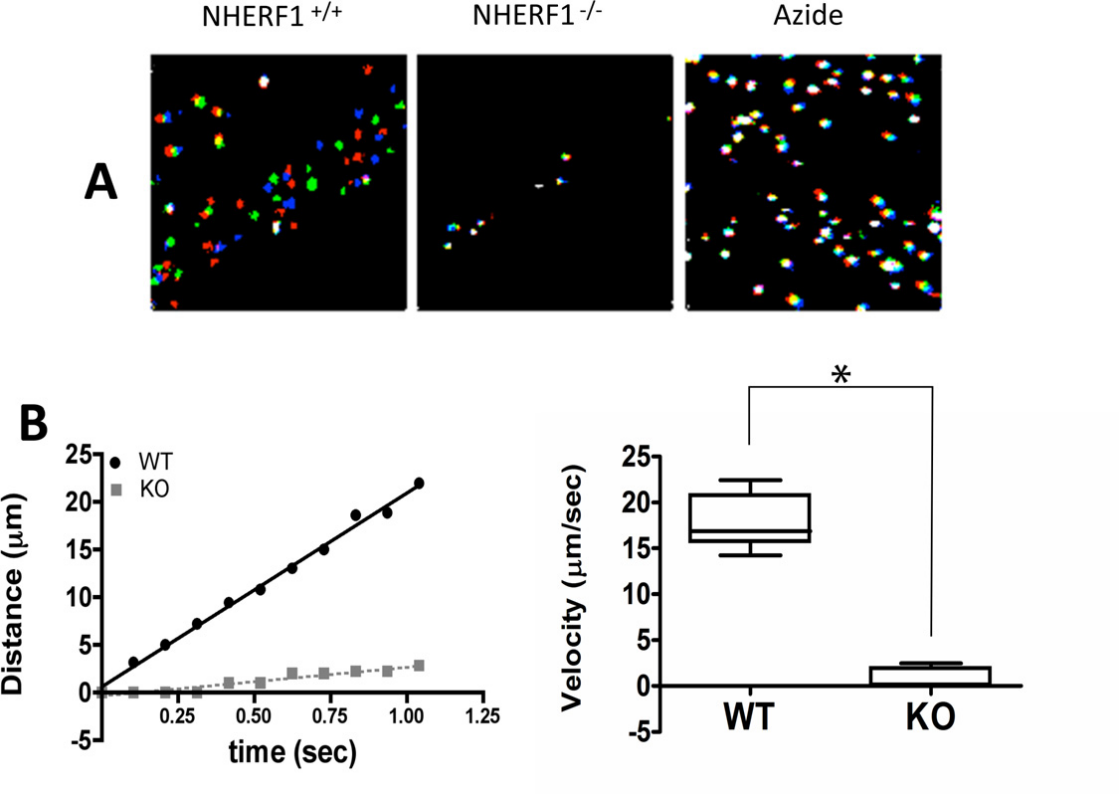
The ependymal cilia of NHERF1^-/-^ mice are dysfunctional. (A) Ciliary function was determined by measuring the motion of 1-μm fluorescein-labeled beads placed within the third ventricle of sagittal brain slices obtained from P28 wild-type and knockout mice. To illustrate the motion, 3 successive images (Δt = 0.1 s) were colored red, green and blue, respectively, and combined in ImageJ. The image on the left, characteristic of the wild-type brain slices, shows no superposition of the three colors, indicating rapid motion of the beads. The beads of the NHERF1^-/-^ slices appear white in the montage because all three colors (red, green and blue) coincide at all times, indicating lack of motion. The right side panel (Azide) shows the results obtained with a wild-type slice poisoned with azide. Refer to S1 Movie for complete visualization of the experiment. (B) Tracking of individual beads in wild-type (WT) and knockout (KO) mice calculated as described. The right panel shows average velocities estimated from the tracking of individual beads. (*) denotes statistically significant differences (p<0.001; results obtained from three independent experiments).

The cross-species hydrocephalus phenotype suggests defects with important functional consequences. Therefore, we examined the ability of the ependymal cilia to generate hydrodynamic fluid movement. This was done using 1-μm fluorescein-labeled latex beads placed onto the third ventricle of freshly prepared brain slices obtained from 28-day old mice (Fig 4*A* and S1 Movie). The fluorescent beads moved rapidly and in an elliptical trajectory in the wild-type slice preparations (average velocity = 18.2 ± 2.2 μm/s; Fig 4*A*). In contrast, there was little discernible movement in the NHERF1^-/-^ slices (average velocity = 1.1±1 μm/s; Fig 4*B*), which was statistically indistinguishable from the Brownian motion of the beads in the azide-poisoned brain slices that served as a negative control (0.81±0.8 μm/s; Fig 4*C*). Interestingly, some beads moved rapidly in the NHERF1^-/-^ preparations, suggesting that ciliary tufts may function independently and that reduced flow is either a consequence of reduced number of cilia or of the lack of organization of the ciliary beat.

**Fig 5:**
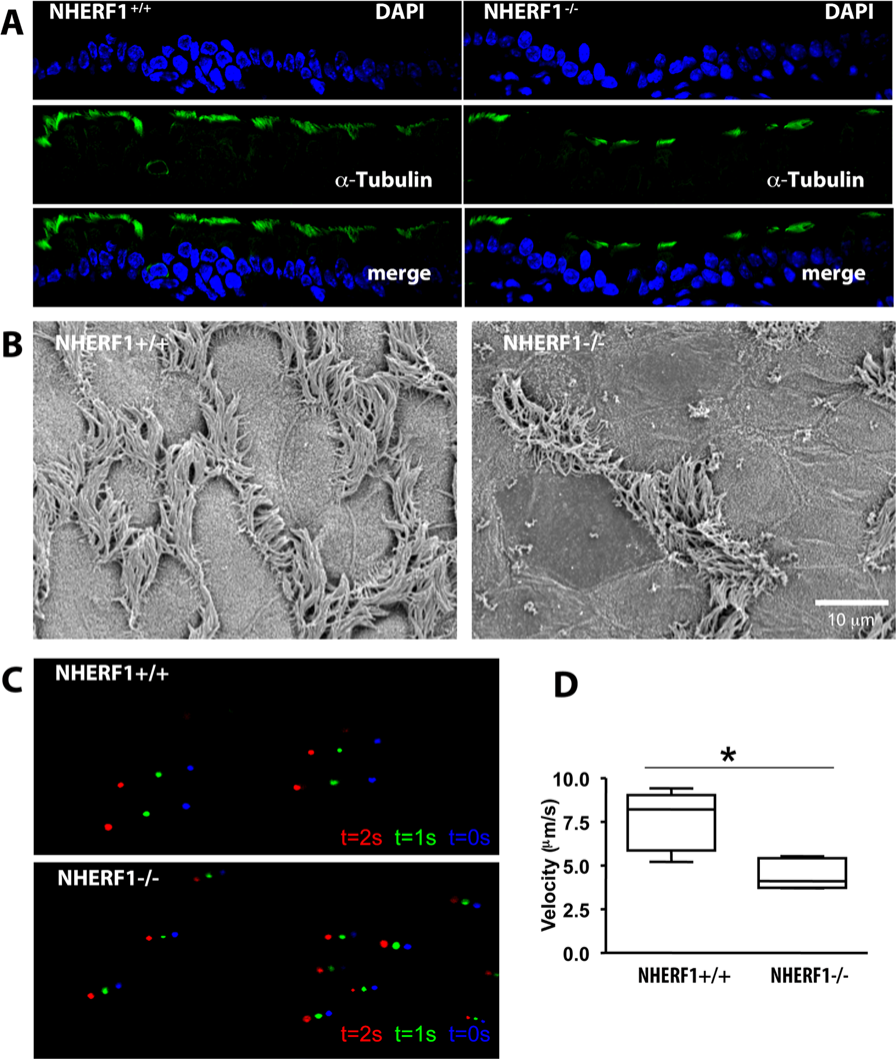
NHERF1 ablation alters cilia organization and function in the respiratory tract. (A) Reduced number of motile cilia on the surface of the respiratory epithelium of NHERF1^-/-^ mice. (B) Scanning electron micrographs of the tracheae of NHERF1^-/-^ mice shows patches of cells with no cilia and cells with vestigial cilia. (C), Ciliary function was determined in sagittal sections of the tracheae of NHERF1^+/+^ and NHERF1^-/-^ animals. Fresh sagittal sections were extended on a microscope slide. After addition of medium and 5 μl of a suspension of fluorescent beads, a coverslip was placed on top of the sections and the movement of the beads was recorded by fluorescence microscopy using a 20X objective at 1-sec intervals. The figure shows the relative positions of the beads using a color code (blue: time = 0, green: time = +1 second, red: time = +2 seconds). (D) The average velocity of the microbeads was determined as described in (C). * denotes statistical significance (p<0.001, N = 3 independent experiments; at least 15 beads tracked per experiment).

To determine whether or not other ciliated organs were affected by NHERF1 ablation, we examined the upper respiratory pathways of wild-type and knockout animals. We observed reduced numbers of cilia and ciliated cells in the trachea of knockout animals, although the defect was significantly less dramatic than in the ependyma. Importantly, the ciliary defects observed in the tracheae of the NHERF1^-/-^ animals were accompanied by a 30% decrease in fluid flow (Fig 5). We conclude, therefore, that NHERF1 plays a general role in the proper orientation and function of motile cilia in multiple tissues and organs.

### NHERF1 regulates Wnt/β-catenin and PCP signaling

Although these results imply that NHERF1 is required for the function of motile cilia, NHERF1 is reportedly excluded from the cilium and is not essential for the formation of primary cilia [21], suggesting that NHERF1 does not play a structural role in cilia formation. Therefore, we turned our attention to the signaling pathways involved in the development of functional motile cilia. A well-documented regulator of motile cilia is the Planar Cell Polarity (PCP) signaling pathway [7, 8]. Ablation of the PCP core genes, Celsr2/3 and Vangl2 causes dysfunction of motile cilia [7, 8]. Importantly, ablation of Celsr2 results in a hydrocephalus phenotype almost identical to the one we describe here [8]. Several of the core PCP genes are potential NHERF1 targets. Eight of the 10 Frizzled genes and both Vangl family members contain canonical Class I PDZ binding motifs in their C-termini. We recently showed that the second PDZ domain (PDZ2) of NHERF1 interacts directly with the C-terminus of a subset of Frizzled receptors [22]. We further showed that NHERF1/Fzd interactions inhibit canonical Wnt signaling in breast cancer cell cultures and in murine breast ducts [22]. Because both Vangl1 and Vangl2 terminate in a canonical Class I PDZ binding motif (-ETSV), we hypothesized that NHERF1 regulates Wnt and PCP signaling in ciliated cells *via* its interactions with Fzd and Vangl proteins. We furthermore hypothesized that the defects caused by NHERF1 ablation are, at least in part, a consequence of altered Wnt and PCP signaling.

**Fig 6:**
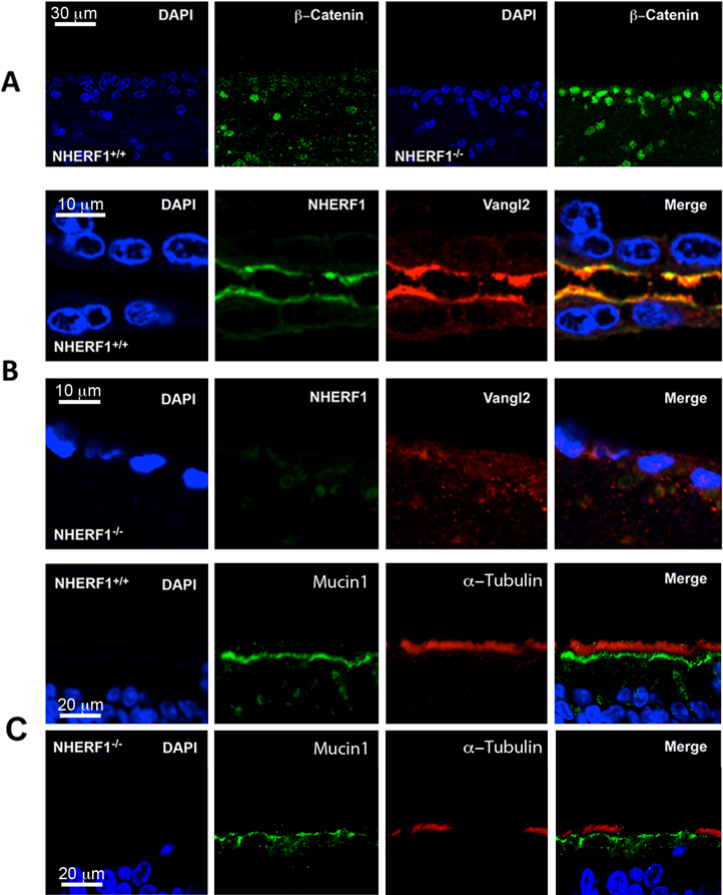
NHERF1^-/-^ mice have altered Wnt signaling and mislocalization of Vangl2. (A) **Increased nuclear β-catenin levels in ependyma of NHERF1**^**-/-**^ **mice.** Brain slices from NHERF1^+/+^ and NHERF1^-/-^ mice were fixed and stained with a monoclonal antibody that recognizes the activated form of β-catenin. (B) **Vangl2 mislocalization in NHERF1**^**-/-**^ **ependyma.** Slices showing the cerebral aqueduct of NHERF1^+/+^ (upper panels) and NHERF1^-/-^ (lower panels) were stained with specific anti-NHERF1 and anti-Vangl2 antibodies. The second row of nuclei is absent in the NHERF1^-/-^ sample because of the dilation of the aqueduct as a consequence of hydrocephalus. (C) **Defective ciliogenesis is not accompanied by gross defects in the apical-basolateral polarization of the ependymal epithelium.** Brain sections showing the third ventricles of wild-type and knockout animals were stained with mucin-1 and α-tubulin antibodies. Notice normal expression of mucin-1 in the ependyma of NHERF1^-/-^ animals independently of the presence of cilia.

We measured nuclear β-catenin levels in brain slices of wild-type and NHERF1^-/-^ animals using specific antibodies. Nuclear β-catenin staining was positive in 78% (±6%) of the knockout ependyma cells and in 8% (±6%) of the wild-type tissues (Fig 6*A*).Because Vangl2 plays a crucial role in the development of tissue polarity, we examined the effects of NHERF1 ablation on the expression and subcellular distribution of Vangl2. In normal ependyma, Vangl2 localizes to the apical surface of the cells in close proximity to the cilia [23]. We found substantial colocalization of NHERF1 and Vangl at the apical surface of the ependyma of wild-type animals (Fig 6*B*). In contrast, there was no apical expression of Vangl2 in the ependyma of the NHERF1^-/-^ mice (Fig 6*B*). Several reports suggested a role for NHERF1 in the establishment of apical-basolateral polarity [10, 11, 24]. Thus, to test whether or not the ablation of NHERF1 caused a general apical-basolateral polarization defect, we determined the distribution of mucin-1, a well-established marker for epithelial polarization that does not contain a PDZ binding motif [25]. As shown in Fig 6*C*, mucin-1 staining was comparable in wild-type and knockout animals. Moreover, the distribution of mucin-1 was very similar in all ependymal cells from the knockout animals independently on whether they contained cilia or not, suggesting that Vangl2 mislocalization is not due to a generic defect in the apical-basolateral polarization of the ependymal epithelium (Fig 6*C*).

### PDZ domain-PDZ ligand interactions regulate the traffic and function of Vangl2

**Fig 7:**
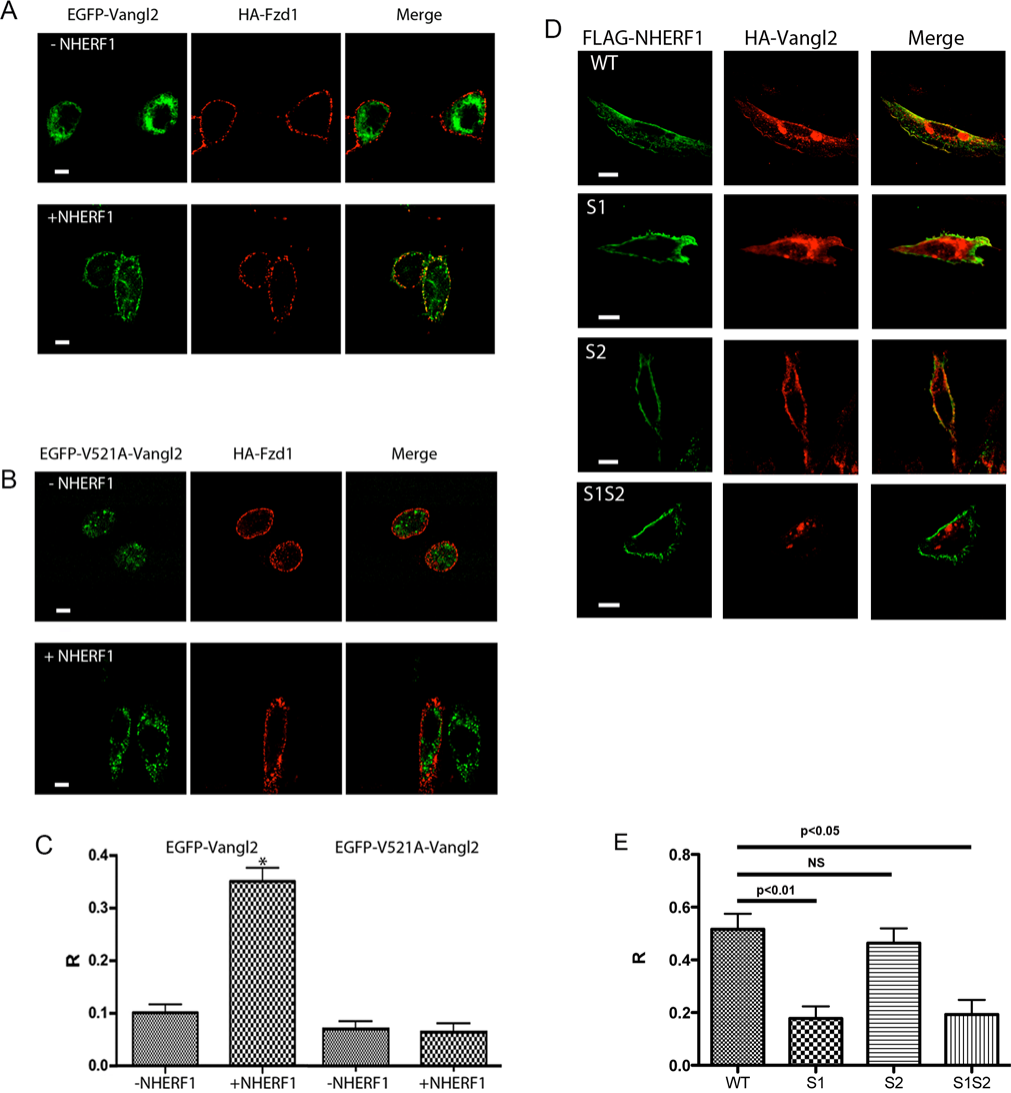
NHERF1 promotes plasma membrane localization of Vangl2. (A) CHO-N10 cells expressing NHERF1 in a tetracycline-sensitive manner were transfected with HA-Fzd1 and EGFP-Vangl2. The cells were treated with 50 nM tetracycline or vehicle, decorated with anti-HA antibody followed by Alexa 594-labeled secondary antibody, and examined by confocal microscopy. (B) Same as A except that the cells were transfected with an EGFP-tagged Vangl2 construct in which the C-terminal PDZ binding motif has been mutated to preclude binding to PDZ domains (V512A-Vangl2). (C) Co-localization of HA-Fzd1 and EGFP-Vangl2. The co-localization was quantified using the Pearson correlation coefficient (R) measured using ImageJ. The correlation coefficients shown represent the average obtained from >50 cells for each set, examined in six separate experiments. The symbol (*) denotes statistically significant differences (p<0.001). (D) NHERF1-induced plasma membrane localization of Vangl2 requires an intact PDZ1. CHO cells co-transfected with Flag-tagged NHERF1 constructs and EGFP-Vangl2 were fixed, decorated with anti-Flag antibodies and examined with a confocal microscope. All NHERF1 constructs were present at the plasma membrane but only the wild-type and S2 NHERF1 constructs co-localized with Vangl2. (D) Quantitative analysis of the co-localization of Vangl2 and NHERF1 mutants. To determine co-localization, we measured the Pearson correlation coefficient of the red and green channels using an ImageJ plugin. The data show the results from >30 cells/set obtained from 4 different experiments. NS: not statistically significant. For all images: the solid white bar is10 μm long.

**Fig 8:**
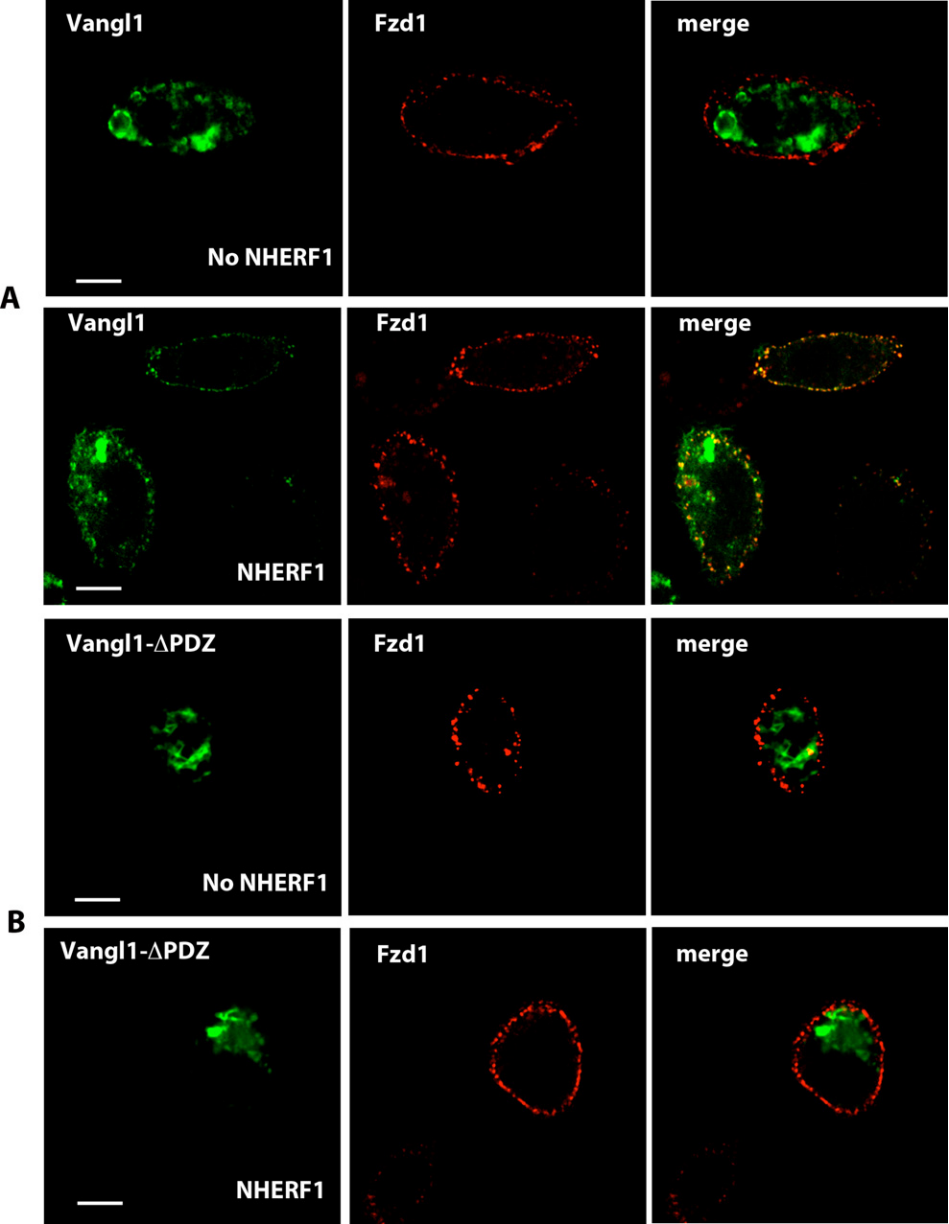
Vangl1 requires NHERF1 to traffic to the plasma membrane. (A) Vangl1 colocalizes with Fzd1 at the plasma membrane only in cells that express NHERF1. (B) A Vangl1 mutant in which the PDZ binding motif has been deleted does not traffic to the plasma membrane independently of the expression of NHERF1. Solid white bar: 10 μm.

The data shown in Fig 6*B* strongly suggest that the traffic of Vangl2 to the apical membrane of ependymal cells is regulated by NHERF1. To further examine this phenomenon, we transfected CHO cells that express NHERF1 in a tetracycline-dependent manner (CHO-N10) [18] with HA-tagged-Fzd1 and EGFP-tagged-Vangl2 constructs. These cells were chosen because, in the absence of tetracycline, the expression of NHERF1 is undetectable. In the absence of NHERF1, the Vangl2 construct was expressed primarily in cytosolic structures (Fig 7*A*). Addition of tetracycline increased the plasma membrane levels of EGFP-Vangl2, where it colocalized with Fzd1, which was used as a plasma membrane marker (Fig 7*A* and 7*C*). To test the hypothesis that NHERF1 regulates Vangl2 localization and traffic by direct interactions with the Vangl2 C-terminal PDZ binding motif, we transfected CHO-N10 cells with EGFP-V521A-Vangl2, a mutant in which the C-terminal valine was replaced by alanine, thus disrupting its binding to PDZ scaffolds [26]. The mutant Vangl2 was retained in cytosolic vesicles, independent of the expression of NHERF1 (Fig 7*B* and 7*C*). Furthermore, equivalent results were obtained with Vangl1, which harbors an identical C-terminal PDZ binding motif (Fig 8). Therefore, the interaction of the PDZ binding motifs of Vangl1/Vangl2 with specific targeting chaperones such as NHERF1 is required for correct localization.

Because NHERF1 contains two PDZ domains, we investigated the effects of mutations of each individual PDZ motif on the distribution of Vangl2. Parental CHO cells (which do not express NHERF1 [27]) were transfected with HA-Vangl2 and Flag-tagged NHERF1 constructs harboring mutations in the core-binding motif of one or both PDZ domains (S1: mutated PDZ1; S2: mutated PDZ2; S1S2: both PDZ domains mutated). All NHERF1 constructs were expressed at or near the plasma membrane of the transfected cells (Fig 7*D*). Vangl2 colocalized with wild-type and S2 NHERF1, but not with the S1 or the S1S2 mutants (Fig 7*D* and 7*E*). Therefore, we conclude that the expression of Vangl2 at the plasma membrane requires the specific interaction of Vangl2 with NHERF1 PDZ1.

**Fig 9:**
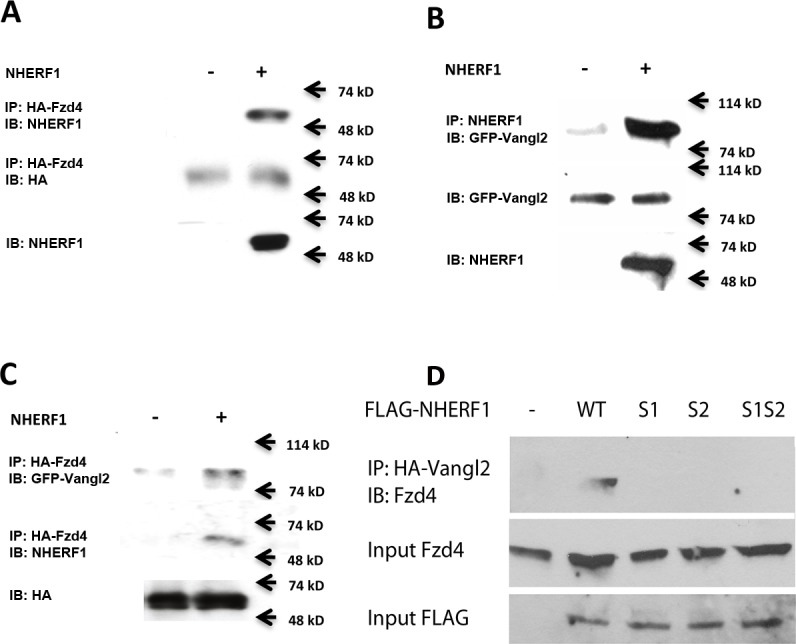
Fzd and Vangl2 bind NHERF1. (A) CHO–N10 cells were transfected with HA-Fzd4, and the formation of a Fzd4-NHERF1 complex was determined by coimmunoprecipitation of the HA-tag followed by Western blotting with specific anti-NHERF1 antibodies. NHERF1 expression was induced by the addition of 100 ng/ml tetracycline. (B) CHO-N10 cells were cotransfected with an EGFP-tagged Vangl2 construct and induced with either vehicle or 100 ng/ml tetracycline. After immunoprecipitation of NHERF1 using a specific antibody, the presence of the GFP label in the immunoprecipitated material was determined by Western blotting. (C) CHO-N10 cells were cotransfected with HA-Fzd4 and EGFP-Vangl2. After induction with either vehicle or 15 ng/ml tetracycline, the HA-tagged material was immunoprecipitated, and the presence of the GFP tag in the co-immunoprecipitate was determined by Western blotting. (D) Both PDZ domains of NHERF1 are required for the interaction of Fzd4 and Vangl2. HEK293S GnTI cells cotransfected with HA-Vangl2 and FLAG-tagged NHERF1 constructs (wt, S1, S2 and S1S2) were lysed and treated with anti-HA antibodies to immunoprecipitate Vangl2.

We previously showed that NHERF1 interacts with Fzd receptors via its PDZ2 domain [22]. Furthermore, some recent data have suggested that the interaction of Vangl2 with Fzd receptors is important for planar cell polarity [8, 28]. Thus, we hypothesized that NHERF1 assembles the formation of Vangl2-Fzd complexes via the interactions of its PDZ domains with these target proteins. To test this hypothesis, we performed coimmunoprecipitation experiments in CHO-N10 cells, which express NHERF1 only in the presence of tetracycline [27], transfected with HA-tagged Fzd4 and EGFP-Vangl2. We found that, as predicted, NHERF1 coimmunoprecipitated with Fzd4 and Vangl2 (Fig 9*A* and 9*B*). We also observed a weak interaction between Vangl2 and Fzd4 that was significantly enhanced by the expression of NHERF1 (Fig 9*C*).

**Fig 10:**
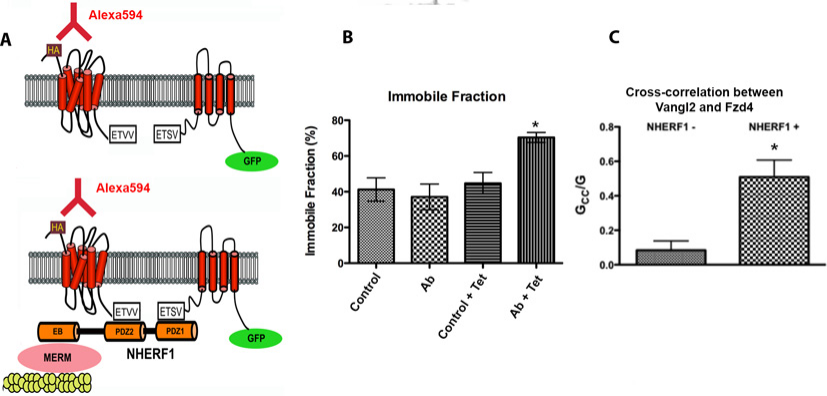
Model for the NHERF1-induced interaction of Fzd4 and Vangl2. (A) Experimental design of the studies described in Panels (B) and (C). We propose that Fzd4 and Vangl2 interact with the PDZ domains of NHERF1. Thus, in the absence of NHERF1, Fzd4 and Vangl2 are expected to interact only weakly; however, NHERF1 expression is predicted to stabilize the formation of Fzd4-Vangl2 complexes. (B) Cells transfected with HA-Fzd4 exposed to the extracellular milieu and EGFP-Vangl2 were treated with an Alexa594-tagged anti-HA antibody. Addition of cross-linking antibodies that recognize the HA tag on Fzd4 immobilizes Vangl2 only when NHERF1 is expressed. CHO-N10 cells transfected with HA-Fzd4 and EGFP-Vangl2 were induced with vehicle or 100 ng/ml tetracycline. Live cells were tagged with Alexa594-labeled anti-HA antibody (HA.11) followed by a secondary anti-mouse IgG cross-linking antibody. The mobility of EGFP-Vangl2 was determined by FRAP. (C) Cross-correlation analysis of the formation of Fzd4-Vangl2 complexes. CHO-N10 cells cotransfected with HA-Fzd4 and EGFP-Vangl2 were incubated with Alexa594-anti-HA antibody. The autocorrelation function of EGFP-Vangl2 and the cross-correlation function of the EGFP label to the Alexa594 label were determined as described [18]. The ratio of the cross-correlation (G_CC_) to the autocorrelation amplitudes is directly proportional to the fraction of Vangl2-Fzd4 complex formed (see[18]). ETVV and ETSV denote the C-termini of Fzd4 and Vangl2, respectively. EB: Ezrin binding domain. MERM: a family of actin-binding protein adaptors that includes moesin, ezrin, radixin and merlin.

To demonstrate that the enhanced formation of Vangl2/Fzd4 complexes in the presence of NHERF1 was a specific consequence of the interactions of the PDZ motifs of NHERF1 with the C-terminal PDZ binding motifs of each target, we co-transfected HEK293S GnTI cells (which contain very low levels of endogenous NHERF1 but express abundant endogenous Fzd4) with HA-tagged Vangl2 and FLAG-tagged NHERF1 constructs (wild-type, S1, S2 and S1S2). We subsequently immunoprecipitated HA-Vangl2 and detected the presence of endogenous Fzd4 in the immunoprecipitate by Western blotting. The results, shown in Fig 9*D*, demonstrate that NHERF1 requires both PDZ domains to promote the formation of Fzd4-Vangl2 complexes. We further studied the formation of these complexes by optical methods using an N-terminal-EGFP-tagged Vangl2 construct and Fzd4 tagged with an HA epitope near the N-terminus such that it is exposed to the extracellular milieu [29] (Fig 10*A*). Cells cotransfected with both constructs were incubated with Alexa594-tagged anti-HA antibody specifically to label the HA-Fzd4 exposed to the membrane. In one set of experiments, the HA-tagged Fzd4 was immobilized at the membrane by addition of a crosslinking secondary antibody [30, 31] and the mobility of the EGFP-Vangl2 construct was determined by FRAP. Fig 10*B* shows that the addition of the crosslinking antibody decreases the mobility of Vangl2 only in cells that express NHERF1. In a second set of studies, we investigated the formation of ternary complexes using image cross-correlation spectroscopy (ICCS), a technique that measures complex formation by determining the correlation of the intensity fluctuations of differentially labeled fluorescent components [18]. In the absence of NHERF1, the Fzd4-Vangl2 cross-correlation was negligible; in contrast, strong positive cross-correlation was observed after induction of NHERF1 expression (Fig 10*C*). These results strongly support the hypothesis that NHERF1 assembles the formation of a complex between Fzd4 and Vangl2.

## Discussion

PDZ proteins are ubiquitous molecular scaffolds that control multiple aspects of protein traffic and function. These regulatory roles are accomplished by the assembly and disassembly of multimeric complexes containing specific signaling and structural molecules that affect the distribution and functional characteristics of the target proteins. NHERF1 is one of the better-characterized members of this family. Initially identified as a regulator of the renal proximal tubule brush border membrane Na^+^/H^+^ exchanger [32], NHERF1 serves an important physiological role in the regulation of mineral-ion homeostasis, including the regulation of parathyroid hormone receptor signaling [17, 27, 33–36]. A global NHERF1 knockout mouse was reported in 2002, and hydrocephalus was noted as a secondary phenotypic characteristic [17]. Our observations confirm the hydrocephalus phenotype of the NHERF1^-/-^ mouse; moreover, we show that *nherf1/slc9a3r1* knockdown in zebrafish embryos also causes hydrocephalus. Furthermore, we report here that the hydrocephalus caused by ablation of NHERF1 is associated with ciliary dysfunction in the ependymal lining of the cerebral ventricles.

How does NHERF1 modulate ciliogenesis? A direct, structural role for NHERF1 in the assembly of the cilium itself is unlikely. NHERF1 is not required for the formation of primary cilia, and is excluded from the cilium [21]. Furthermore, the ependymal cells of NHERF1^-/-^ animals contain some cilia with normal 9+2 structures, and of normal length and diameter. In addition, our data show that the absence of NHERF1 does not alter the apical-basolateral polarization of Mucin-1, a protein that does not contain a PDZ binding motif (Fig 5C), suggesting that the ciliogenesis defect observed in the NHERF1^-/-^ mice is unlikely to result from a generalized loss of apical-basolateral polarity in the ependyma. Comparison of the NHERF1^-/-^ phenotype with other forms of hydrocephalus strongly suggests that the problem lies in a defective PCP signaling pathway. Mice missing the PCP genes Celsr2 and Celsr3 develop hydrocephalus linked to defective motile cilia [8]. In particular, the Celsr2^-/-^ mice present reduced numbers of ciliated ependymal cells, reduced numbers of cilia in individual tufts, and defective cilia organization, which results in impaired hydrodynamic CSF flow trhat strongly resembles the NHERF1^-/-^ phenotype [8]. Furthermore, the SEM and TEM images of the ependymal surface of NHERF1^-/-^ and Celsr2^-/-^ mice are remarkably similar. In both animal lines, the ependymal defects are accompanied by altered traffic of the basal bodies to the cell surface, as shown by the significant fraction of embedded basal bodies observed in the knockout animals (Fig 2E and [8]). This phenomenon may be a consequence of the impaired traffic of the basal bodies to the surface followed by degradation of ectopic basal bodies [8], but this remains to be demonstrated.

Recent studies suggest that the docking of basal bodies to the cell surface of primary mouse airway cells and the subsequent assembly of motile cilia requires the temporal association of an apical web-like actin structure [37]. Planar cell polarity genes, such as Celsr2 and Vangl2, play an important role in the remodeling of the actin cytoskeleton that accompanies the organization of motile cilia [38]. It is reasonable to propose that the defective organization of ependyma cilia caused by NHERF1 ablation is, at least in part, a consequence of the effects of NHERF1 on the distribution and cell surface expression of Vangl2 reported in this study. However, it is important to point out that NHERF1 interacts directly with cytoskeleton-associated proteins, such as ezrin [10, 13, 39] and α-actinin-4 [40]. Since these protein targets also regulate cytoskeletal dynamics, these interactions may contribute to the overall role of NHERF1 in the organization of motile cilia. However, we did not observe global disruptions of the actin cytoskeleton in the NHERF1^-/-^ mice. Ependyma cells developed normal apical-basolateral polarity as judging from the distribution of mucin-1 and intestinal microvilli appear normal in the NHERF1^-/-^ animals (not shown), suggesting normal apical-basolateral organization.

Notably, both NHERF1 and the Celsr proteins play an important role in the regulation of the plasma membrane distribution of Vangl2. However, the phenotypes associated with the disruption of Vangl2 (ablation, *looptail* mutations) are characterized by serious neural issues such as craniorachischisis and other severe neural tube defects [41], a phenotype not observed in NHERF1^-/-^ or Celsr2/3^-/-^ animals. The origin of the different phenotypes is not well understood, but it may be related to redundancy and the restricted patterns of expression of the redundant genes.

An interesting finding reported here is the increased β-catenin signaling resulting from NHERF1 ablation in ependymal cells. This is consistent with our earlier observations of increased nuclear β-catenin in the mammary tissue of NHERF1^-/-^ mice. However, no well-established links between increased β-catenin signaling and impaired ciliogenesis have been reported. Thus, and largely based on the great similarity between the NHERF1 and Celsr knockout phenotypes, we propose that the hydrocephalus phenotype of the NHERF1^-/-^ mouse is caused primarily by defective PCP resulting from the mislocalization of Vangl2 in the ependyma. Importantly, since NHERF1^-/-^ tissues also exhibit increased β-catenin levels, we further propose that NHERF1 serves as a molecular router that regulates the relative input of Fzd-mediated signals to these alternative pathways: 1) inhibition of Wnt/β-catenin signaling [22]; and 2) recruitment of Vangl proteins to the plasma membrane to promote PCP signaling. This latter role of NHERF1, *viz* regulating traffic of target proteins to the plasma membrane of polarized epithelia, is consistent with previous studies [11, 13, 42, 43].

The direct regulation of Wnt signaling pathways by specific PDZ proteins has been documented. Many of the molecular components of the Wnt signaling pathways contain canonical PDZ domains or C-terminal PDZ-binding motifs, including 8 of the 10 mammalian Fzd receptors, β-catenin, and Vangl1/2 [44]. However, little is known about the role of these PDZ binding motifs and their interacting partners in the regulation of Wnt and PCP downstream signals. Because PDZ proteins are essentially scaffolds, the formation of multimeric complexes is a central feature of their role in the regulation of biological processes. Thus, the multi-PDZ protein MAGI-3 has been implicated in the stabilization of complexes containing Vangl2 and Fzd4/Fzd7 that localize to cell-cell contact sites [45]. We show here a similar scaffolding role for NHERF1 although with a very different subcellular distribution. NHERF1 is targeted to the apical end of polarized cells [13]. Inasmuch as our results demonstrate that Vangl2 is apical in wild-type but not in NHERF1^-/-^, we conclude that NHERF1 is required for the apical localization of Vangl2 in ependyma.

The formation of Fzd-Vangl2 complexes has been postulated to play a central role in PCP signaling [7, 8, 28]. In mammals, Vangl2 interacts with Fzd3 [28], but evidence for the interaction of Vangl2 with other members of the Fzd family is lacking. We show here that the N-terminal PDZ domain of NHERF1, PDZ1, binds the C-terminus of Vangl2. Likewise, the C-terminal PDZ domain of NHERF1, PDZ2, interacts with a subset of Fzd receptors that includes Fzd4 [22]. It is, therefore, not surprising that NHERF1 promotes the interaction of Vangl2 with Fzd4 by acting as a scaffold linking both proteins. This scaffolding function is likely to hold for other members of the Fzd family that contain PDZ binding motifs in their C-termini. For example, we show that NHERF1 expression substantially increases the co-localization of Vangl1 and Vangl2 with Fzd1 (see Fig 7 and Fig 8). Thus, this model provides a mechanism for the regulation of PCP events by Fzd receptors that, unlike Fzd3, do not interact efficiently with Vangl2. Furthermore, this role of NHERF1 may not be unique. Recent reports suggest that other multi-PDZ proteins may also assemble protein complexes that include Fzd receptors and Vangl proteins [45]. Thus, PDZ scaffolds may play redundant roles in coupling PCP to diverse downstream functions depending on their distribution and relative levels of expression in specific cells and tissues. Our data so far demonstrate that NHERF1 is necessary for the correct organization and function of motile cilia.

## Supporting Information

S1 Fig **A:** D. *rerio* embryos injected with Splice-MO develop hydrocephalus (48 h post- fertilization). **B:** Posterior crista of the otic vesicle of a *D*.*rerio* embryo injected with Splice-MO. Ac-tubulin: acetylated tubulin. The phalloidin stain highlights the apical localization of actin.
(TIF)

S2 Fig S100β-staining of NHERF1^+/+^ and NHERF1^-/-^ ependyma shows that the ependymal cells of the NHERF1^-/-^ animals develop normally. (TIF)

S1 Movie Ciliary function was evaluated using brain sections exposing the third ventricle. Fresh sections were mounted on a microscope slide and immersed in MEM. An aliquot (2 μl) of a fluorescent bead suspension was added within the cavity of the ventricle and the movement of the beads was examined using a 20X water immersion objective mounted on an Olympus microscope. Images were acquired at 1 second intervals. The video shows the motion of the beads in sections obtained from wild-type and NHERF1 knockout animals. The panel labeled “wild-type + azide” shows the Brownian motion of the beads in a slide containing a wild-type section poisoned with azide to stop all ciliary motion. Refer to the legend of Fig 4.
(AVI)
