# Supplementary figures and images for: The PDZ Protein Na+/H+ Exchanger Regulatory Factor-1 (NHERF1) Regulates Planar Cell Polarity and Motile Cilia Organization
Source: PLoS One. 2016 Apr 7;11(4):e0153144. doi: 10.1371/journal.pone.0153144 (PMC4824468; doi:10.1371/journal.pone.0153144)

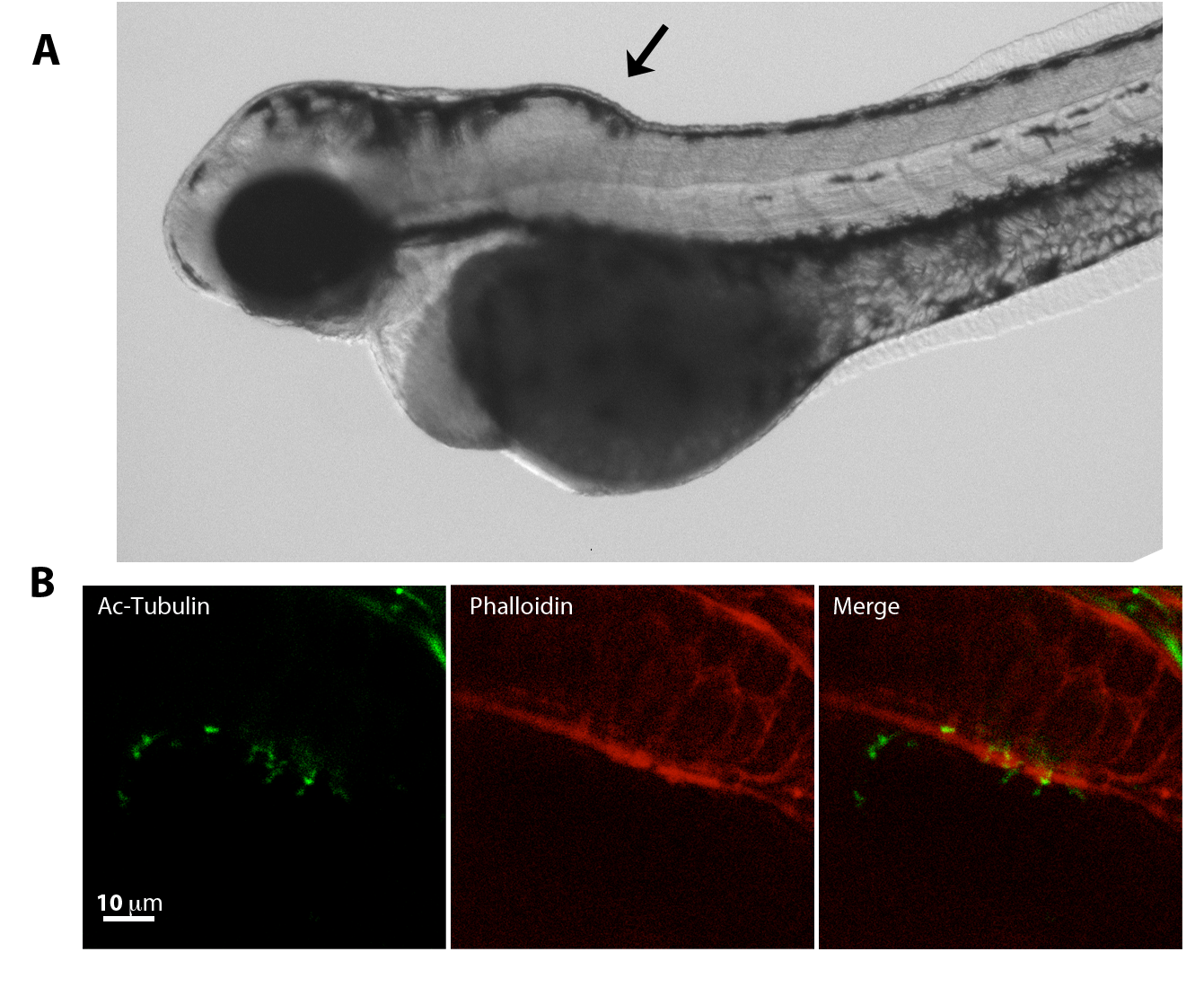

Supplement: S1 Fig — A: D. rerio embryos injected with Splice-MO develop hydrocephalus (48 h post- fertilization). B: Posterior crista of the otic vesicle of a D.rerio embryo injected with Splice-MO. Ac-tubulin: acetylated tubulin. The phalloidin stain highlights the apical localization of actin. (TIF) [file pone.0153144.s001.tif]

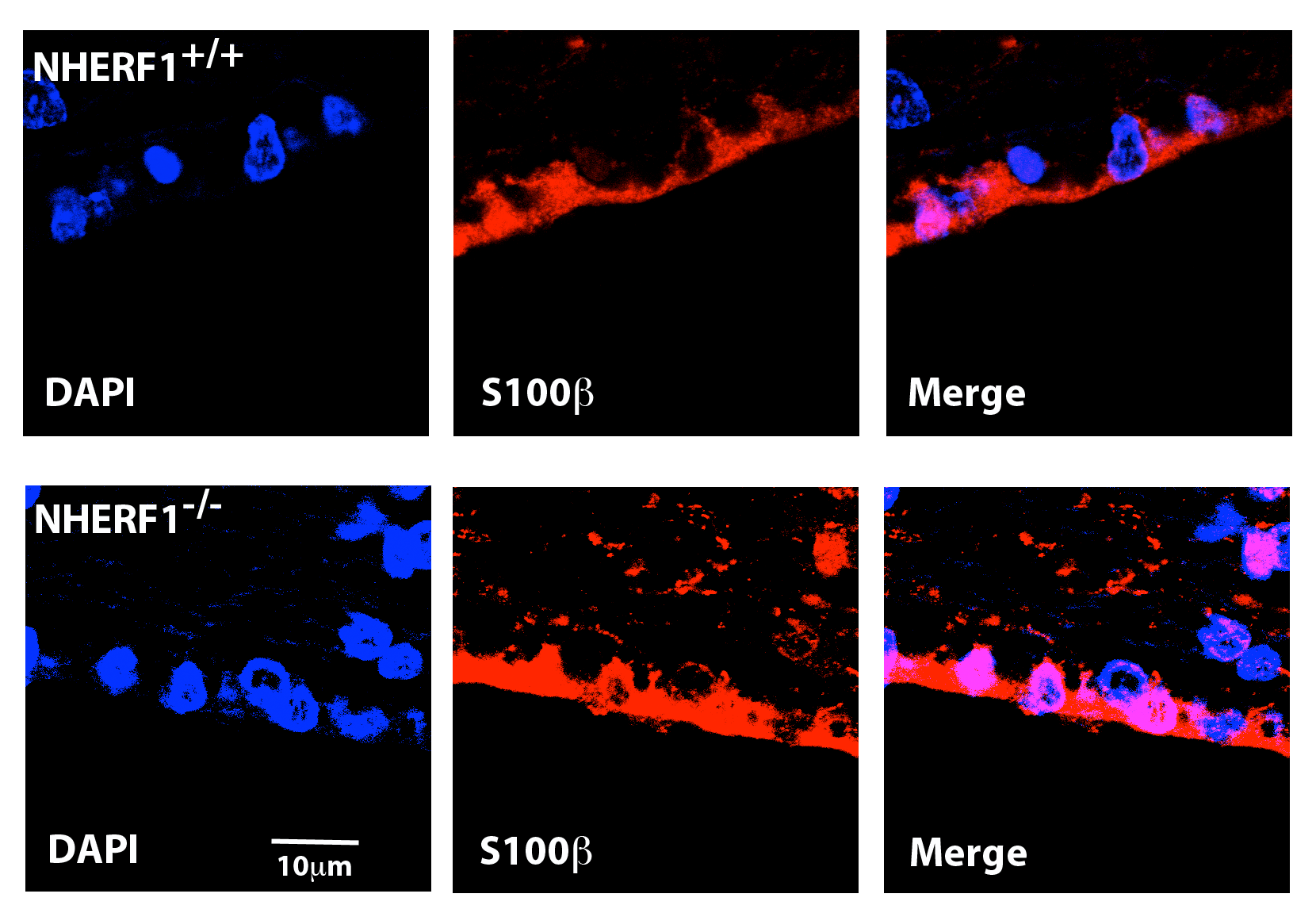

Supplement: S2 Fig — (TIF) [file pone.0153144.s002.tif]
